# TLR4 mediates post-traumatic depression via kynurenine pathway activation in a murine traumatic brain injury model

**DOI:** 10.3389/fphar.2026.1744031

**Published:** 2026-04-15

**Authors:** Chang-Hong Li, Bo Wang, Ruo-Bing Gao, Yan Peng, Ke Chen, Xiao-Hui Li, Ping Li

**Affiliations:** 1 Department of Anatomy, Anhui Medical University, Hefei, Anhui, China; 2 The Molecular Biology Center, State Key Laboratory of Trauma and Chemical Poisoning, Department of Army Occupational Disease, Daping Hospital, Army Medical University (Third Military Medical University), Chongqing, China; 3 Center of Bone Metabolism and repair, Laboratory for Prevention and Rehabilitation of Training injuries, State Key Laboratory of Trauma and Chemical Poisoning, Trauma center, Research Institute of Surgery, Daping Hospital, Army Medical University (Third Military Medical University), Chongqing, China; 4 Laboratory of Stem Cell and Tissue Engineering, Chongqing Medical University, Chongqing, China

**Keywords:** delayed depression, kynurenine pathway (KP), TLR4, traumatic brain injury (TBI), treatment

## Abstract

**Introduction:**

Traumatic brain injury (TBI) is a major risk factor for major depressive disorder (MDD), yet the underlying mechanisms remain poorly defined. This study demonstrates that toll-like receptor 4 (TLR4) activation drives depressive-like behaviors through dysregulation of the kynurenine pathway (KP) in a murine model of moderate TBI.

**Methods and Results:**

Using male C57BL/6J mice subjected to controlled cortical impact, we observed depression-like phenotypes (reduced sucrose preference, prolonged immobility in forced swimming tests) specifically at 28 days post-TBI, with an incidence of 28.13%. Proteomics and immunofluorescence analyses revealed significant upregulation of hippocampal TLR4 expression and signaling pathway activation, concomitant with microglial activation. Crucially, the TLR4-specific inhibitor TAK-242 (administered i.p. from days 21–28 post-TBI) ameliorated depressive behaviors and suppressed phosphorylation of NF-κB p65. Mechanistically, TBI induced microglia-dependent upregulation of key KP enzymes indoleamine 2,3-dioxygenase 1 (IDO1) and kynurenine monooxygenase (KMO), leading to accumulation of the neurotoxic metabolite quinolinic acid (QUIN). This TLR4-KP axis was validated *in vitro*: LPS-stimulated BV2 microglia showed increased IDO1/KMO/QUIN expression, which was abolished by TAK-242 pretreatment.

**Conclusion:**

Our findings establish a novel TLR4–KP–QUIN pathway as a critical mediator of post-TBI depression, providing a mechanistic basis for TLR4-targeted therapies. In addition to neuroinflammatory effects, this work mainly mediates dysregulation of TBI-related neuropsychiatric sequelae through metabolism, highlighting TLR4 inhibition as a promising strategy for mitigating chronic depressive outcomes after brain injury.

## 1 Introduction

Traumatic brain injury (TBI), a leading global cause of death and disability, poses a significant public health burden (Maas et al., 2022). Despite this, the long-term consequences of TBI remain incompletely understood. Clinical focus has historically prioritized acute life-saving interventions and mid-term motor/cognitive recovery, often overlooking severe psychiatric sequelae—particularly the established link between TBI and major depressive disorder (MDD) (Al-Kader et al., 2022; Ryttersgaard et al., 2022). Notably, even moderate TBI (mTBI; e.g., concussion) substantially elevates lifetime MDD risk (Uiterwijk et al., 2022). Current treatments for post-TBI depression include pharmacotherapy and multimodal approaches (e.g., cognitive behavioral therapy, exercise rehabilitation). However, randomized controlled trials show inconsistent outcomes: some antidepressants (e.g., SSRIs like sertraline or citalopram) demonstrate symptomatic benefits, while others (e.g., tricyclics) show negligible efficacy versus placebo or induce adverse effects (e.g., seizures) (Cui L. et al., 2024; Dobrek and Głowacka, 2023; Liu et al., 2024; Liu et al., 2019). This therapeutic uncertainty underscores the poorly defined pathophysiology of post-TBI MDD, necessitating urgent investigation into its underlying mechanisms.

Post-TBI depression is increasingly recognized as a multifactorial disorder involving structural damage, neurotransmitter dysregulation, neuroinflammation, hypothalamic-pituitary-adrenal (HPA) axis disruption, and impaired neuroplasticity (Cui L. et al., 2024). Among these, neuroinflammation has emerged as a critical mediator, contributing to depression via cytokine release, microglial/astrocytic activation, neurotransmitter imbalances, HPA dysregulation, and neural network disruption (Kohler et al., 2016; Liu et al., 2024). Consequently, targeting neuroinflammation represents a promising therapeutic strategy for post-TBI MDD.

Toll-like receptor 4 (TLR4) serves as a key sentinel in sustaining chronic neuroinflammation. TBI-induced blood-brain barrier compromise and tissue damage release endogenous damage-associated molecular patterns (DAMPs) that act as TLR4 ligands, triggering persistent activation (Karimy et al., 2020). TLR4 signaling occurs primarily through two pathways: the MyD88-dependent pathway (driving NF-κB activation and pro-inflammatory cytokine transcription (e.g., interleukin-1β, IL-1β, tumor necrosis factor-α, TNF-α, and IL-6) and the TRIF-dependent pathway (inducing interferon responses). Together, these pathways perpetuate a self-sustaining inflammatory cascade (Kim et al., 2023; Liu et al., 2014; Zhang et al., 2022b). Preclinical studies indicate that TLR4-knockout mice or TLR4 antagonists (e.g., TAK-242) reduce neuroinflammation, mitigate brain damage, and improve behavioral outcomes post-TBI (Chen et al., 2021; He et al., 2020; Zhang et al., 2014). Critically, TLR4 overactivation is increasingly implicated in depression pathogenesis (Figueroa-Hall et al., 2020), suggesting its relevance as a therapeutic target for post-TBI MDD—though further validation is warranted.

Notably, TLR4 signaling may regulate the kynurenine pathway (KP), which generates neuroactive metabolites (Fila et al., 2021). For instance, following intracerebral hemorrhage, TLR4 signaling upregulates the key KP enzyme indoleamine 2,3-dioxygenase 1 (IDO1) in the brain via the p38-MAPK pathway, thereby contributing to disease progression (Cao et al., 2025; Zhu et al., 2024). Similarly, in schizophrenia, TLR4 activation induces KP pathway activity, leading to abnormal accumulation of kynurenic acid (KYNA) and quinolinic acid (QUIN), which is implicated in cognitive impairment (Yang et al., 2026). Discussion under neuroinflammatory conditions, Microglia-derived KP metabolites (e.g., quinolinic acid [QUIN], 3-hydroxykynurenine [3-HK]) exhibit neurotoxicity via excitotoxicity, oxidative stress, and mitochondrial dysfunction. Post-TBI neuroinflammation likely disrupts KP balance, promoting accumulation of these neurotoxic compounds and contributing to neurodegeneration and depressive phenotypes (Meier and Savitz, 2022; Wang et al., 2018). Clinical evidence further underscores the significance of the KP in depression, demonstrating that a genetic mutation in IDO2 increases depression risk after childhood maltreatment (Sun et al., 2025), and elevated QUIN levels correlate with higher depression incidence in both general TBI and sports-related concussion patients (Meier et al., 2020; Yan et al., 2015). Thus, key questions remain: whether TLR4 modulates KP after TBI, and whether this interaction drives post-TBI depression.

In this study, we employed a murine TBI model to investigate TLR4’s role in post-TBI depression and its interplay with the KP. Depressive-like behaviors were assessed using standardized behavioral tests. Immunofluorescence, quantitative proteomics, and molecular techniques revealed upregulated TLR4 expression and pathway activation. Administration of the TLR4 inhibitor TAK-242 ameliorated depressive behaviors and downregulated key KP enzymes (IDO, KMO) and metabolites (quinolinic acid), as confirmed by WB, qPCR, and ELISA. Lipopolysaccharide-stimulated (LPS) microglia further demonstrated TLR4-KP pathway coordination, supporting TLR4-mediated KP activation. These findings elucidate a novel TLR4–KP axis in post-TBI depression, providing a mechanistic foundation for TLR4-targeted therapies.

## 2 Methods

### 2.1 Animals

Male C57BL/6 J mice aged 6–8 weeks were used in the experiments; they were acquired from the Experimental Animal Center of Army Medical Center. The mice were group-housed in cages under pathogen-free conditions, with *ad libitum* access to food and water. They were kept in a laboratory room maintained at a temperature of 21 °C ± 1 °C and a humidity of 52% ± 2%, under a 12-h light/12-h dark cycle (except during the stress exposure period). Prior to the start of the experiments, the mice were allowed a 2-week acclimation to adapt to the new environment (Wang et al., 2020). Every animal experiment was carried out strictly in accordance with the Army Military Medical University’s rules and ethical standards (AMUWEC20210248).

### 2.2 TBI model

The automatic impact machine (TBI-0310, PSI, United States) was used to build the mouse model of TBI as previously reported. In short, mice were put in a stereotaxic frame and given sodium pentobarbital (3 mg/kg, i.p.) to induce anesthesia (Lian et al., 2017; Zhang N. et al., 2025). Over the right parietal cortex, lateral to the sagittal suture, a craniectomy with a diameter of 4 mm was carried out. To produce a degree of moderate to mild TBI, an impact was created using a 3 mm flat-tip impounder at a speed of 3.5 m/s, a depth of 1.5 mm, and a dwell duration of 500 ms (Du et al., 2022; Miao et al., 2025). With the exception of the effect, sham-operated animals received the same treatments. The mice were put on an electric heating blanket to recuperate from the anesthesia following surgery, and then they were put back in their cage.

### 2.3 Treatments

Three experimental groups of mice were randomly assigned: Three experimental groups of mice were randomly assigned: one for traumatic brain damage (TBI + vehicle), one for TAK242 treatment of TBI (TBI + TAK242), and one for Sham. TAK242 was administered starting on day 21 post-TBI and continuing until day 28 in compliance with established experimental protocols. The TAK-242 group was given intraperitoneal injections of TAK-242 (3 mg/kg/day i.p. HY-11109/CS-0408, MedchemExpress) (Gong et al., 2018; Jiang et al., 2020; Ono et al., 2020). In addition, IDO 1/TDO-in-4/GSK180 (20 mg/kg/day i.p. HY-151108, HY-112179 medchemex Express) was also given to the key enzyme inhibitors of IDO and KMO respectively (Mole et al., 2016; Zhang et al., 2022a). To create a functional solution, all drugs are freshly reconstituted in corn oil every injection day. Before administration, the solution was incubated at 37 °C for 2 hours to provide sufficient dissolution and stability.

### 2.4 Behavioral tests

Following TBI induction, neurological function scores were assessed at 1, 3, 7, 14, and 28 days post-injury. Additionally, behavioral tests were conducted according to the following schedule: the sucrose preference test (SPT) on days 13 and 27, the forced swimming test (FST) on days 14 and 28, and the open field (OFT) and elevated plus maze tests (EPM) on days 15 and 29.


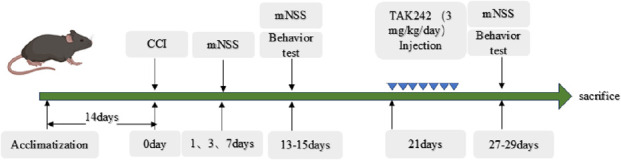


#### 2.4.1 modified Neurological Severity Score (mNSS) test

Neurological severity scoring of mice was performed at 24 h, 3 days, 7 days, 14 days, and 28 days post-traumatic brain injury (TBI) following previously established protocols (Supplementary Figure 1). Neurological functions, including motor and sensory function, reflexes, and balance, were evaluated using the modified Neurological Severity Score (mNSS) (Ho et al., 2024). The mNSS ranges from 0 to 18 points, where a score of 0 indicates normal neurological status, and higher scores represent more severe deficits. Specifically, scores of 1–6 points correspond to mild injury, 7–12 points to moderate injury, and 13-18 points to severe injury (Yang et al., 2022).

#### 2.4.2 Open field test (OFT)

The test mice were individually placed into an open-field arena (45 × 45 cm) to assess spontaneous locomotor activity. Each mouse was gently positioned in the center of the arena, and its behavior was recorded over a 5-min session using a video tracking system (Noldus EthoVision XT, version EV115) (Du et al., 2022). The total distance moved, as well as the time spent in the central zone (20 × 20 cm) were automatically quantified and analyzed by the software (Noldus EthoVision XT, version EV115).

#### 2.4.3 Sucrose preference test (SPT)

Before the sucrose preference test, each mouse was housed separately and allowed to get acquainted to the testing environment. Mice were pre-exposed to a 1% sucrose solution for 2 weeks during the habituation period, with bottle placements being changed every day (Wang et al., 2018). Mice were deprived of water for 24 h following adaption. The mice were then allowed to drink ordinary water and sugar solution for 1 hour. The bottles were weighed to determine fluid consumption, and sucrose preference was calculated as sucrose intake divided by total fluid intake × 100% (Wang et al., 2020).

#### 2.4.4 Forced swimming test (FST)

The forced swimming test was performed in a transparent glass cylinder (height30 cm, diameter 15 cm), which was filled with fresh water (22 °C ± 1 °C) up to a height of 25 cm from the bottom. Each session lasted 5 min and was recorded on video for subsequent analysis (Noldus EthoVision XT, version EV115) (Cui M. et al., 2024). The duration of immobility was measured during the final 4 min of the trial. Immobility was defined as the cessation of active escape-directed behaviors, characterized by floating motionless with only minimal movements necessary to keep the head above the water surface.

#### 2.4.5 Elevated plus maze (EPM) test

The EPM test was used to assess anxiety-like behavior in mice. The apparatus consisted of two open arms (30 × 8 × 0.5 cm) and two enclosed arms (30 × 8 × 20 cm), arranged perpendicularly and connected by a central open platform (8 × 8 cm). Each mouse was placed individually onto the central area facing an open arm and allowed to explore the maze freely for 5 min. After each trial, the maze was thoroughly cleaned with 75% ethanol to remove olfactory cues. The data were collected using Noldus Ethovision XT 12.1 software (Noldus Information Technology, Wageningen, Netherlands) (Chen X. et al., 2023; Du et al., 2024).

### 2.6 Proteomics detection

This work examined the function of TLR4 in traumatic brain injury (TBI) using a label-free quantitative proteomics approach. The experimental groups’ hippocampal tissues were gathered, and tryptic digestion and protein extraction were then performed. A nano-liquid chromatography-tandem mass spectrometry (nano-LC-MS/MS) apparatus for data collecting was then used to examine the resultant peptides (Xia et al., 2021; Zhao et al., 2022). Protein identification and label-free quantification were achieved by processing the raw mass spectrometry data through database searches. Differentially expressed proteins (DEPs) were then filtered using strict criteria (|Fold Change| > 1.5 and p-value <0.05) (Xu et al., 2025). In order to determine their underlying biological functions, the list of DEPs was then sent to the Majorbio Cloud platform for KEGG pathway enrichment analysis.

### 2.7 Cell culture and treatment

BV2 microglial cells were obtained from the Shanghai FUHENG Biotechnology Corporation (FuHeng Cell Center, Shanghai,China). Biosharp supplied the LPS (*Escherichia coli* 055: B5, # BS904-10 mg), which was used in this experiment at a dose of 1 μg/mL. The dosage employed in this investigation was 10 nM (dissolved in DMSO) of TAK-242 (item number HY-11109), which was purchased from MedChem Express. The cells were grown at 37 °C in a 5% CO_2_ incubator using high-glucose DMEM that contained 10% fetal bovine serum, 1% penicillin, and streptomycin (Zhang et al., 2019). Following 80% cell growth and fusion, cells were pretreated with 10 nM TAK-242 for 24 h, followed by a 24-h treatment with or without LPS (1 μg/mL) (Xie et al., 2023).

### 2.8 Reverse transcription and real-time quantitative RT-PCR

Total RNA was extracted from hippocampal tissue for 28 days and microglial cells using a commercial RNA isolation kit (Promega, LS1040) (Du et al., 2022). RNA purity and concentration were determined by measuring the absorbance at 260 nm and 280 nm using a spectrophotometer (NanoDrop One UV-Vis). Reverse transcription was carried out with the GoScript™ Reverse Transcription System (Promega, A2790) under the following conditions: incubation at 25 °C for 5 min, followed by 42 °C for 50 min, and enzyme inactivation at 70 °C for 15 min. The resulting complementary DNA (cDNA) was stored at 4 °C until further use. Quantitative PCR (qPCR) was performed using GoTaq® qPCR Master Mix (Promega) and gene-specific primers (listed in Table 1), in accordance with the manufacturer’s protocol. The amplification conditions consisted of an initial denaturation step at 95 °C for 10 min, followed by 40 cycles of denaturation at 95 °C for 15 s and annealing/extension at 55 °C for 1 min.

**TABLE 1:** Sequences of target genes in this study.

Gene name (house mouse)	​	Primer sequence (5′ to 3′)	Length
GAPDH	Forward	AGG​TTG​TCT​CCT​GCG​ACT​TCA	21
	Reverse	TGG​TCC​AGG​GTT​TCT​TAC​TCC	21
TLR4	Forward	GTA​AAG​CTA​AGC​GGC​CTA​AT	20
	Reverse	AGG​GCC​CTA​TTG​GCG​TTA​AGC	21
IDO	Forward	AGG​GCC​CTA​TTG​GCG​TTA​AGC	20
	Reverse	TCC​CAG​ACC​CCC​TCA​TAC​AG	20
KMO	Forward	ATG​GCA​TCG​TCT​GAT​ACT​CAG​G	22
	Reverse	CCC​TAG​CTT​CGT​ACA​CAT​CAA​CT	23
KAT2	Forward	GGA​GAA​GAG​GGT​TTC​CTG​GC	20
	Reverse	CAG​CTT​TGG​GAA​CAT​GCC​AC	20

### 2.9 western blot (WB) analysis

Proteins were extracted from hippocampal tissues and cells as follows: 30 mg of tissue and cells (5 × 106 cells/well) were homogenized in 0.3 mL of RIPA lysis buffer (Nantong Botai Institute of Biotechnology) supplemented with 1 mM protease inhibitors and phosphatase inhibitors (Ma et al., 2024). Samples were incubated on ice for 30 min and then centrifuged at 12,000 × g for 15 min at 4 °C; the resulting supernatant was collected. The protein concentration of the supernatant was determined using an enhanced BCA Protein Assay Kit (cat. no. P0013; Nantong Botai Institute of Biotechnology, Nantong, China) according to the manufacturer’s instructions. Protein extracts were separated by 10% sodium dodecyl sulfate-polyacrylamide gel electrophoresis (SDS-PAGE) and transferred to polyvinylidene difluoride (PVDF) membranes. The membranes were blocked with 5% nonfat dry milk prepared in Tris-buffered saline with Tween-20 (TBST; containing 0.1% Tween-20) and then incubated overnight with the respective primary antibodies (details in Table 2). After washing with TBST, the membranes were incubated with secondary antibodies for 1 h at room temperature. Protein bands were visualized using an enhanced chemiluminescence (ECL) kit and quantified by densitometric analysis using ImageJ software. The internal loading control was β-actin.

**TABLE 2:** Primary and secondary antibodies used in this study.

Primary antibody	Dilution	Source	Brand	Cat. #	Secondary antibody	Dilution	Brand	Cat. #
TLR4 (WB)	1:1000	Mouse	CST	RT1666	Goat anti- mouse IgG, HRP-linked antibody Goat anti-rabbit IgG, HRP-linked antibody	1:3000 1:3000	CST CST	7074S
β-ACTIN	1:2000	Rabbit	Selleck	C21N23				
Ido1	1:1000	Rabbit	Proteintech	13268-1-AP				
KMO	1:1000	Rabbit	Proteintech	10689-1-AP				
KAT2	1:2000	Mouse	Proteintech	66575-1-AP				
GAPDH	1:5000	Rabbit	Selleck	A19L10				
TLR4 (IF)	1:100	Rabbit	ThermoFisher	PA564939	Goat anti-rabbit IgG H&L (Cy3®) preadsorbed	1:200	Abcam	ab6939
Ido1	1:200	Rabbit	Proteintech	13268-1-AP	Goat anti-mouse IgG H&L (alexa Fluor® 488)	1:300	Abcam	ab150113
KMO	1:100	Rabbit	Proteintech	10689-1-AP				
KAT2	1:200	Mouse	Proteintech	66575-1-AP	Horse anti-mouse IgG, HRP-linked antibody	1:3000	CST	7076S
IBA1	1:500	Guinea pig	Oasis biofarm	OB-PGP055	Goat Anti-Guinea pig IgG H&L (alexa Fluor® 647)	1:200	Abcam	ab150187

WB: Western blotting, IF: immunofluorescence.

### 2.10 Immunofluorescence (IF) staining

Experiments on behavior were carried out 28 days after TBI. Mice were sedated with sodium pentobarbital (3 mg/kg, intraperitoneal injection [i.p.]) after finishing the experiment, and their hearts were then perfused with 4% paraformaldehyde and normal saline. In order to produce coronal sections (25–30 μm) for immunofluorescence, free-floating sections were cleaned in PBS, permeabilized for 20 min with 0.3% Triton X-100, and blocked for 30 min with either goat serum or 3% bovine serum albumin (BSA) (Cen et al., 2023). Following an overnight incubation with primary antibodies (details are shown in Table 2), sections were rinsed in PBS and treated for 1 hour at 37 °C with fluorescently tagged secondary antibodies. DAPI was used to counterstain the nuclei (Cat: C0065, Solarbio).

For cells: Overnight, cells were cultivated at 37 °C with 5% CO_2_. Following adhesion, cells were stimulated with LPS, treated with TAK-242, and then incubated for an additional 24 h. Cells were permeabilized with 0.5% Triton X-100 for 10 min, blocked with 5% BSA for 30 min, then fixed with 4% paraformaldehyde for 20 min (Peng et al., 2024). The following procedures were the same as for tissue sections. A Leica TCS-SP2 confocal microscope (488, 543, 633 nm laser lines) was then used to obtain high-magnification images after coverslips had been mounted with anti-fluorescence quenching medium. Each group’s average fluorescence intensity was measured.

### 2.11 Enzyme-linked immunosorbent assay (ELISA)

For quinolinic acid (QUIN) determination, it obtained mouse brain tissues and cell supernatant. According to the guidelines included with the ELISA kits for QUIN CB10886-Mu20268) (Keaibo, Shanghai, China), QUIN levels were determined. A microplate reader was used to measure each sample’s absorbance (OD value) at 450 nm.

### 2.12 Statistical analyses

GraphPad Prism software (Version 9.3.0) was used to analyze and graphically depict all of the data. One-way or two-way analysis of variance (ANOVA) was used to compare groups, and the proper *post hoc* tests were used for pairwise comparisons. The mean ± standard deviation (SD) was used to express each variable. The 2^(-ΔΔCt)^ approach was used to examine quantitative real-time polymerase chain reaction (RT-qPCR) data. Statistical significance was defined as a probability value (P-value) of less than 0.05.

## 3 Results

### 3.1 TBI induces depression-like behaviors (behavioral assessments)

**FIGURE 1:**
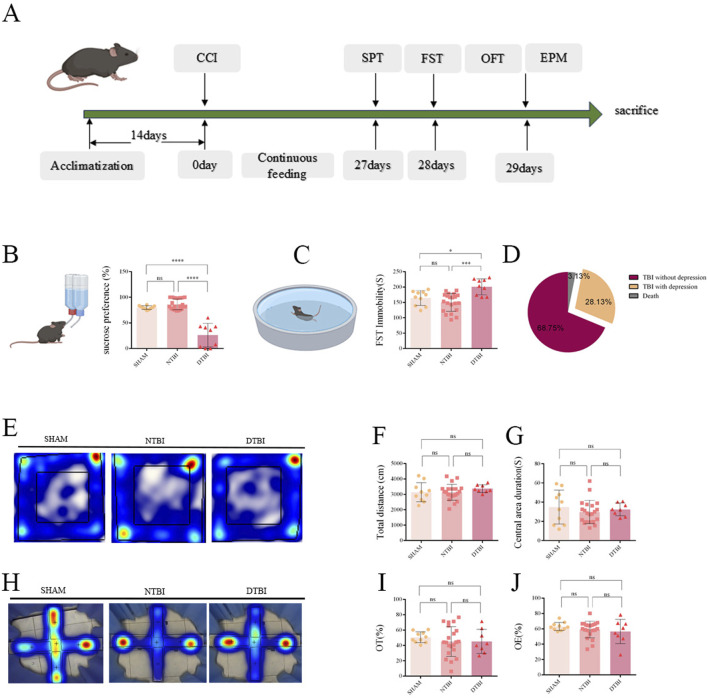
Depressive-like behaviors in mice 28 days post-TBI. **(A)** Timeline of behavioral assessments: Sucrose Preference Test (SPT), Forced Swimming Test (FST), Open Field Test (OFT), and Elevated Plus Maze Test (EPM) conducted at 28 days post-TBI. **(B)** SPT: DTBI mice showed a significantly decreased percentage preference for sucrose solution (One-way ANOVA by Tukey’s *post hoc* test, n = 10, 21, 9; F _(2, 37)_ = 62.16, P < 0.0001). **(C)** FST: DTBI mice exhibited significantly increased immobility time (One-way ANOVA by Tukey’s *post hoc* test, n = 10, 21, 9; F _(2, 37)_ = 10.83, P = 0.0002). **(D)** Prevalence of depressive-like behaviors in TBI mice was approximately 28.13%. **(E–G)** OFT: No significant differences were observed in total distance moved **(E,F)**; One-way ANOVA, F _(2, 36)_ = 0.68, P = 0.51) and time spent in the center zone **(E,G)**; One-way ANOVA, F _(2, 36)_ = 0.56, P = 0.58). **(H–J)** EPM: time spent in the open arms **(H,I)**; One-way ANOVA, F _(2, 36)_ = 0.4013, P = 0.6724) and Both number of entries into the open arms **(H,J)**; One-way ANOVA, F_(2, 36)_ = 0.4606, P = 7921) showed no significant difference. (Data analyzed by one-way ANOVA followed by Tukey’s *post hoc* test; n = 10, 21, 8; *P < 0.05, ***P < 0.001, ****P < 0.0001; ns: no significance; CCI: controlled cortical impact; NTBI: non-depressed TBI; DTBI: depressed TBI; OE: open arm entries; OT: time in open arms).

The modified Neurological Severity Score (mNSS) for all groups remained below 6.5, confirming a moderate-intensity TBI model. Compared to controls, TBI mice exhibited significantly elevated mNSS at 24 h post-injury, which returned to baseline levels by days 14 and 28 (Supplementary Figure 1). Then, we assessed whether mTBI induces depressive-like behavior at 14 or 28 days post-injury (Figure 1A).

Firstly, we confirmed that there were no significant differences in spontaneous locomotor activity in the open field test (OFT, Figures 1E–G) or anxiety-like behaviors in the elevated plus maze (EPM, Figures 1H–J) between depressed TBI (DTBI) and non-depressed TBI (NTBI) mice, thus excluding confounding effects of TBI-related motor impairment and anxiety. In the SPT, mice in the DTBI group exhibited a markedly reduced sucrose preference ratio compared with the sham group; in contrast, no significant difference was observed between the NTBI group and the sham group. Consistently, the FST showed the same trend, with DTBI mice displaying significantly prolonged immobility time relative to sham, further validating the establishment of a depressive-like phenotype. In the TBI mouse model, mice exhibiting depression-like phenotypes were identified based on a dual-behavioral criterion: reduced sucrose preference in the (SPT) combined with increased immobility time in the FST (Figures 1B,C). Specifically, mice with sucrose preference below a composite cut-off (control baseline – 2 SD) in the SPT were preliminarily screened, and those that also showed prolonged immobility in the FST compared to controls were classified as depressive (DTBI). Using this approach, we observed a 28.13% incidence of depression-like behavior in DTBI mice at 28 days post-TBI (Figure 1D). Notably, no significant differences in sucrose preference or FST immobility were detected at 14 days compared to the sham group (Supplementary Figure 2), suggesting that depression-like behavior in this model emerges by 28 days after TBI.

### 3.2 Proteomics reveals high expression of TLR4 and related target genes in hippocampus of mice 28 days after traumatic brain injury

**FIGURE 2:**
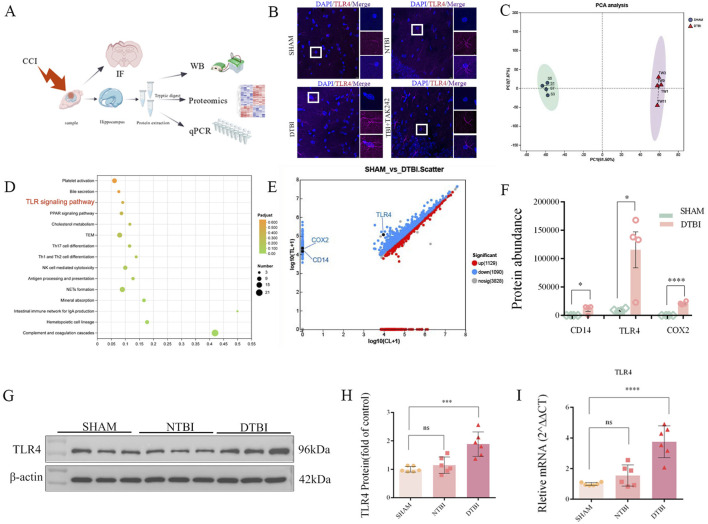
TLR4 upregulation in the hippocampus 28 days post-TBI. **(A)** Detection methods for TLR4 changes: immunofluorescence (IF), proteomics, Western blot (WB), and qPCR. **(B)** IF analysis: Enhanced TLR4 immunofluorescence intensity (red) with morphological alterations in the hippocampal nuclei (DAPI, blue; scale bar = 25 µm). **(C–F)** Proteomics analysis: activation of the TLR4 pathway (unpaired t-test, t = 3.335, df = 6, P = 0.0157) and elevated expression of its downstream genes Cd14 (unpaired t-test, t = 2.993, df = 6, P = 0.0242) and Cox2 (unpaired t-test, t = 15.08, df = 6, P < 0.0001). **(G–I)** WB and qPCR: increased TLR4 expression in the DTBI group (WB: one-way ANOVA, F _(2,15)_ = 14.63, P = 0.0003; qPCR: F_(2,15)_ = 24.26, P < 0.0001; data analyzed by one-way ANOVA with Tukey’s *post hoc* test; n = 6 per group; *P < 0.05, ***P < 0.0001).

To elucidate the role of TLR4 in post-TBI depression (Figure 2A), IF analysis revealed robustly elevated TLR4 expression in the hippocampus of TBI with depression (DTBI) mice compared to controls and NTBI (Figure 2B). This upregulation was concomitant with microglial activation, evidenced by increased cell density and morphological shifts toward an amoeboid phenotype (Supplementary Figure 3). Then, both TLR4 protein and mRNA levels were quantified significant increases in DTBI group via WB (Figures 2G, H) and qPCR (Figure 2I) analyses, which was consistent with the aforementioned behavioral results (Figures 1B,C). To further elucidate the role of TLR4 in post-TBI depression, we performed quantitative proteomic analysis via mass spectrometry (MS) on hippocampal tissues from the sham and diffuse traumatic brain injury (DTBI) groups (Figures 2C–F). Proteins in the DTBI group with a p-value <0.05 and a fold change >1.5 compared with the sham group were included in subsequent analyses. A total of 6047 proteins were differentially expressed in this set, among which 1129 were upregulated and 1090 were downregulated. Principal component analysis (PCA) in Figure 2C demonstrated good intragroup clustering and a significant intergroup distance between the 2 mouse groups. Additionally, Kyoto Encyclopedia of Genes and Genomes (KEGG) database enrichment analysis revealed activation of the TLR4 pathway and its upstream and downstream associated proteins among the upregulated proteins in the DTBI group (Figures 2D–F). Collectively, these data establish that TLR4 pathway activation contributes to the pathogenesis of post-TBI depressive-like.

### 3.3 Behavioral evaluation of mice in each group after intervention

**FIGURE 3:**
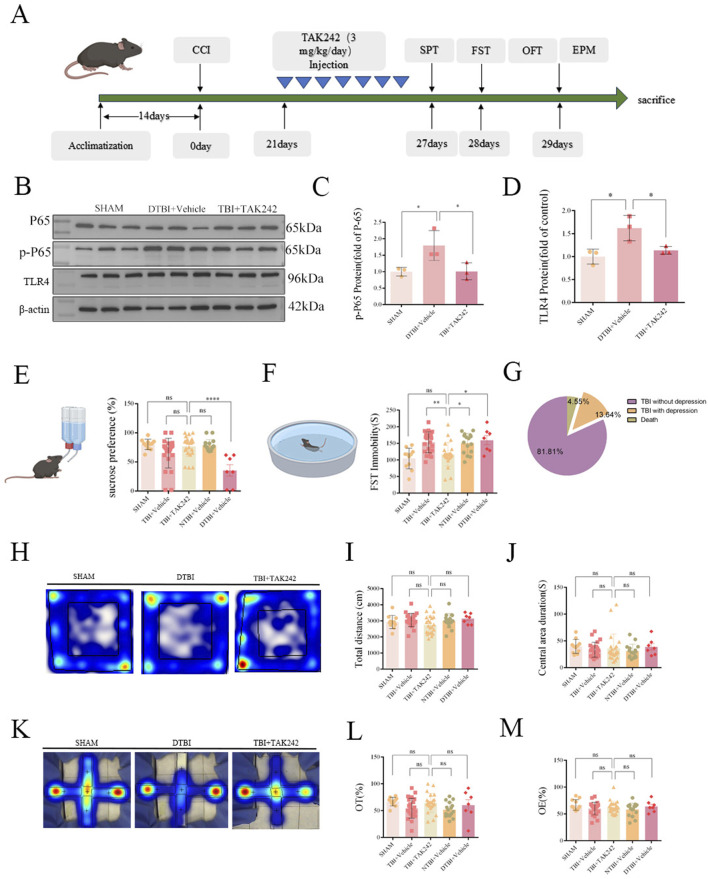
TAK242 (TLR4 inhibitor) attenuates TBI-induced depressive-like behaviors. **(A)** Behavioral assessments: SPT, FST, OFT, and EPM. **(B–D)** WB: Reduced TLR4 and p-P65 expression in TBI + TAK242 group (TLR4: F (2,6) = 8.703, P = 0.017; p-P65: F (2,6) = 6.464, P = 0.032). **(E)** SPT: Increased sucrose preference (F (4,75) = 7.187, P < 0.0001). **(F)** FST: Decreased immobility time in TAK242-treated mice (F (4,75) = 7.713, P < 0.0001). **(G)** Prevalence of depressive-like behaviors in TBI mice by treated was approximately 13.64%. **(H–J)** OFT: No differences in total distance moved **(I)**: F (4,75) = 1.688, P = 0.1615) or center duration **(J)**: F (4,75) = 0.5221, P = 0.7198). **(K–M)** EPM: No changes in open arm time **(L)**: F F (4,75) = 2.292, P = 0.0674), or entry in open arms **(M)**: F (4,75) = 1.143, P = 0.3430). (Data analyzed by one-way ANOVA with Tukey’s *post hoc* test; n = 11, 23, 23, 16, 7; *P < 0.05, ****P < 0.0001).

To further validate TLR4’s role in post-TBI depressive-like behaviors, we intraperitoneally administered the specific TLR4 inhibitor Resatorvid (TAK-242) for 7 consecutive days, beginning 21 days post-TBI (Figure 3A). Western blot analysis demonstrated that TAK-242 treatment significantly suppressed hippocampal TLR4 expression and abolished NF-κB p65 phosphorylation compared to vehicle-treated DTBI (Figures 3B–D), corroborated by reduced TLR4 immunofluorescence (Figure 2B). Concomitant behavioral assessments demonstrated that TLR4 inhibition: Reduced despair-like behaviors and improving anhedonia. As shown in Figure 3E, although there was no statistical significance in the TBI + TAK-242 group compared with the TBI + Vehicle group, the trend clearly indicated a therapeutic effect of the TBI + TAK-242 group. Furthermore, the TBI + TAK-242 group showed a significant statistical difference compared with the DTBI + Vehicle group, with significantly increased sucrose preference (Figure 3E), which also strongly indicated that anhedonia was improved after TAK-242 administration. Notably, in Figure 3F, we observed that the TBI + TAK-242 group significantly shortened the immobility time in FST compared with the traumatic brain injury (TBI)+Vehicle group, further confirming the efficacy of the TLR4 inhibitor. To rule out confounding factors, TLR4 inhibition did not cause changes in locomotor activity (OFT total distance, center time; Figures 3H–J) or anxiety-like behaviors (EPM open arm entries/durations; Figures 3K–M). Collectively, these results establish that TLR4 activation critically contributes to the development of depressive-like behavior following TBI (Figure 3G).

### 3.4 TLR4 mediates activation of the neurotoxic KP branch in TBI-induced depression, reversed by TAK-242

**FIGURE 4:**
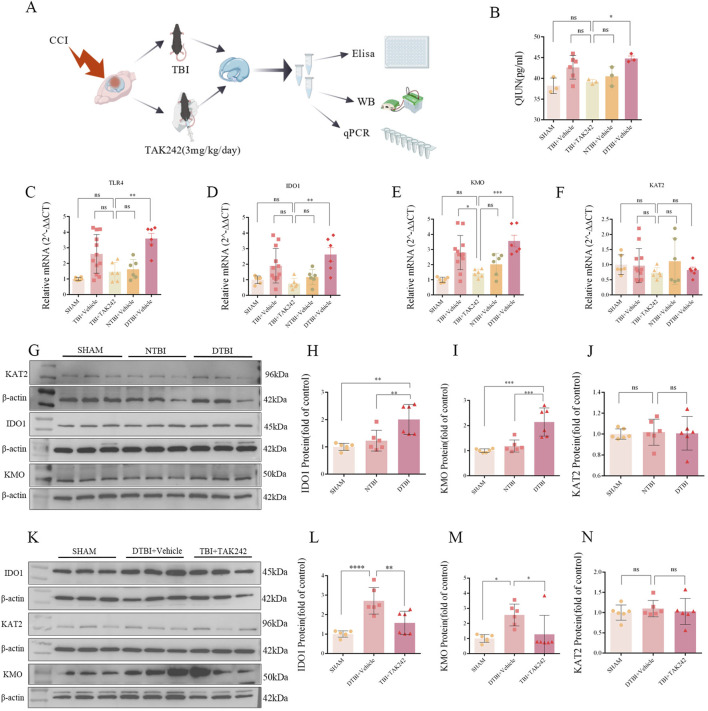
Dysregulation of kynurenine pathway (KP) enzymes post-TBI and TAK242 effects. **(A)** Detection methods: ELISA, qPCR, WB; All data normalized to control (set as 1.0). **(B)** ELISA: Increased QUIN post-DTBI; reduced by TAK242 (F _(4,13)_ = 4.888, P = 0.0126). **(C–F)** qPCR: Upregulated TLR4 **(C)**: F _(4,31)_ = 8.836, P < 0.0001), IDO1 **(D)**: F _(4,31)_ = 5.339, P = 0.0022), and KMO **(E)**: F _(4,31)_ = 9.928, P < 0.0001); no change in KAT2 **(F)**: F _(4,31)_ = 0.6002, P = 0.6653). **(G–J)** WB: Increased IDO1 **(H)**: F _(2,6)_ = 9.981, P = 0.012) and KMO **(I)**: F _(2,6)_ = 27.01, P = 0.001); unchanged KAT2 **(J)**: F _(2,6)_ = 0.025, P = 0.976). **(K–N)** TAK242 effects: Reduced IDO1 **(L)**: F _(2,6)_ = 15.47, P = 0.004) and KMO **(M)**: F _(2,15)_ = 5.749, P = 0.0140); no KAT2 change **(N)**: F _(2,6)_ = 0.226, P = 0.804). (Data analyzed by one-way ANOVA with Tukey’s test; n = 6 (WB), n = six (qPCR); *P < 0.05, **P < 0.01, ***P < 0.001, ****P < 0.0001; IDO1: indoleamine 2,3- dioxygenase 1, KMO: kynurenine monooxygenase; KAT2: kynurenine aminotransferase 2; QUIN: quinolinic acid).

In order to reveal the potential mechanism of depression-like behavior in mice after TBI, the changes of KP pathway core enzymes IDO1 (indoleamine 2,3- dioxygenase 1), KMO (kynurenine monooxygenase) and KAT2 (kynurenine aminotransferase 2) were detected by qPCR and WB (Figure 4A) after 28 days of TBI (DTBI and NTBI). qPCR showed that compared with the control group and NTBI group, the expression levels of IDO1 and KMO in DTBI group increased, but KAT2 did not change significantly (Figures 4C–F); WB also shows similar results (Figures 4G–J). Consequent quinolinic acid (QUIN or QA) accumulation was observed only in DTBI group (Figure 4B). These findings confirmed that DTBI selective activation of the KP’s neurotoxic arm. Moreover, inhibition of TLR4 abolished this KP dysregulation: TAK-242 treatment significantly reduced IDO1 (Figures 4D,K,L), KMO (Figures 4E,K,M), and QUIN (Figure 4B) levels in DTBI mice, but KAT2 did not change significantly (Figure 4N).

To investigate the relationship between the KP and post-TBI depression, we pre-administered key KP enzyme inhibitors—IDO1/TDO-in-4 (an IDO inhibitor) and GSK180 (a KMO inhibitor)—via intraperitoneal injection to mice and detected their behaviors. Our data revealed that inhibition of IDO and KMO reduced the incidence of TBI-induced depression to 14.29% and 7.14%, respectively, which was consistent with the results obtained following TLR4 inhibition (Supplementary Figures 4E,J. Furthermore, OFT (Supplementary Figures 4F, 5K) and EPM (Supplementary Figures 4G,L) assays excluded the confounding effects of the mice’s general health status and anxiety-like behaviors. Moreover, QUIN, a KP’s toxic metabolite, also exhibited changes consistent with the depressive-like behaviors (Supplementary Figure 4B). Collectively, these results not only further demonstrate that the KP is one of the pathways through which TLR4 modulates depression following TBI, but also reveal that TLR4 activation mediates post-TBI depression-like behavior by selectively upregulating the neurotoxic KP branch (IDO1 → KMO → QUIN, Figure 7).

### 3.5 LPS stimulation increased IDO1 and KMO levels in microglia, while TAK242 inhibited their increase

**FIGURE 5:**
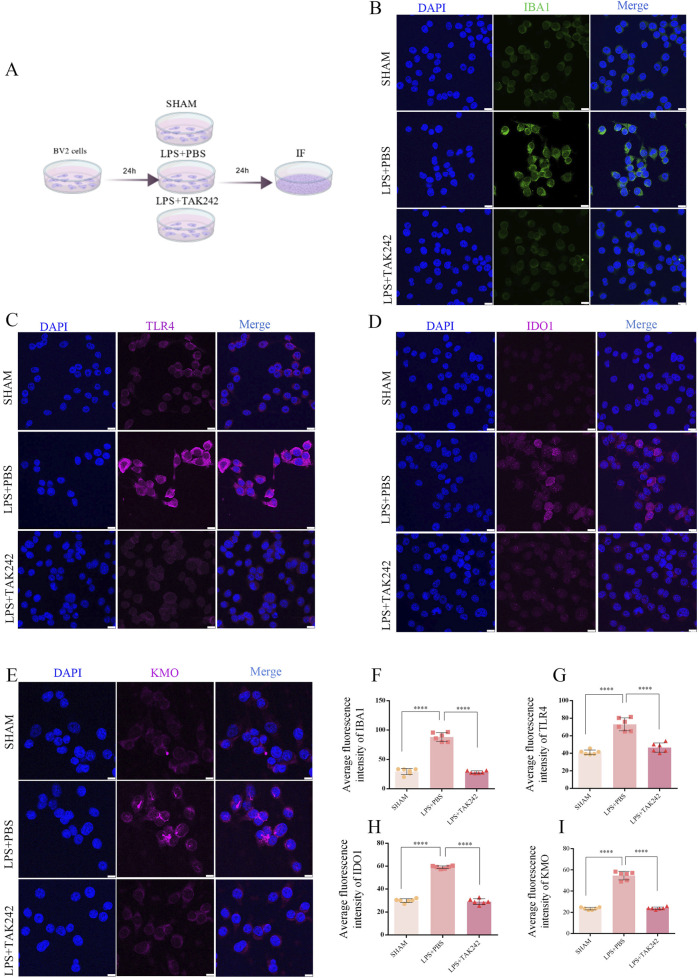
LPS activates kynurenine pathway in BV2 cells. **(A)** IF staining for TLR4, IBA1, IDO, and KMO (63× magnification). **(B–I)** Quantification: Increased intensity of IBA1 **(B,G)** F _(2,15)_ = 231.9, P < 0.0001), TLR4 **(C,F)** F _(2,15)_ = 59.35, P = 0.0001), IDO1 **(D,H)** F _(2,15)_ = 508.8, P < 0.0001), and KMO **(E,I)** F _(2,15)_ = 312.3, P < 0.0001). (One-way ANOVA with Tukey’s test; n = 6 per group; ****P < 0.0001; scale bar = 25 µm).

To further investigate whether TLR4 regulates the KP through IDO1 and KMO, we employed a microglial culture model (Figure 5A). This approach was based on our preliminary histochemical findings of microglial activation in DTBI mice (Supplementary Figure 3), which is consistent with the reports from other labs (Cui et al., 2020). IF analysis confirmed that LPS stimulation specifically induced the accumulation of Iba1 (Figures 5B,G), TLR4 (Figures 5C,F), IDO1 (Figures 5D,H), and KMO (Figures 5E,I) in microglia at 24 h. This LPS-induced upregulation was significantly suppressed by treatment with the TLR4 inhibitor TAK242 (Figures 5B–I).

**FIGURE 6:**
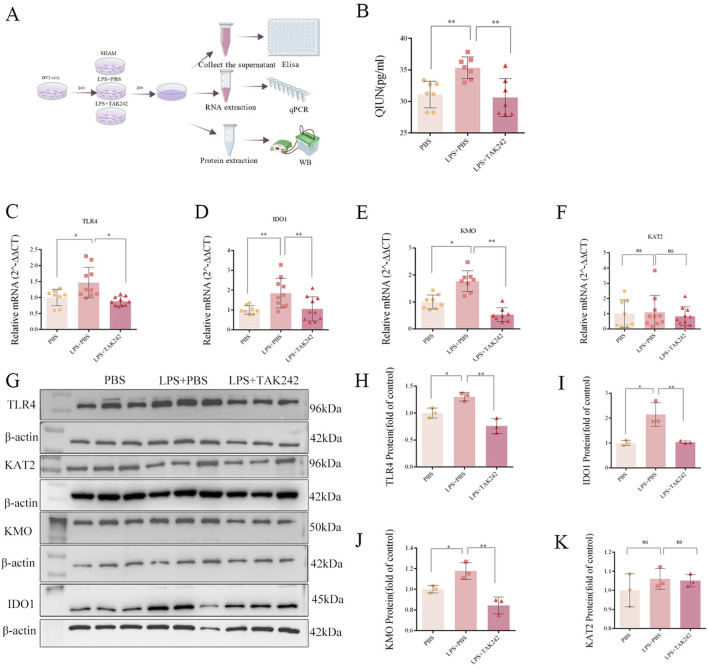
TAK242 blocks LPS-induced kynurenine pathway activation. **(A)** Detection methods: ELISA, qPCR, WB. Data normalized to control 1.0. **(B)** ELISA: QUIN elevation post-LPS (F _(2,18)_ = 8.514, P = 0.003); attenuated by TAK242. **(C–F)** qPCR: Upregulated TLR4 **(C)**: F _(2,25)_ = 8.673, P = 0.0014), IDO1 **(D)**: F _(2,25)_ = 6.198, P = 0.007), KMO **(E)**: F _(2,21)_ = 33.31, P < 0.0001); no KAT2 change **(F)**: F _(2,25)_ = 0.224, P = 0.801). **(G–K)** WB: TAK242 reduced TLR4 **(G,H)** F _(2,6)_ = 19.73, P = 0.002), IDO **(G,I)** F _(2,6)_ = 15.85, P = 0.004), KMO **(G,J)** F _(2,6)_ = 17.26, P = 0.003); unchanged KAT2 **(G,K)** F _(2,6)_ = 0.821, P = 0.484). (Data analyzed by one-way ANOVA with Tukey’s test; n = 7 (ELISA), 8–10 (qPCR), 3 (WB); *P < 0.05, **P < 0.01, ****P < 0.0001).

Consistent with these findings, qPCR and Western blot analyses (Figure 6A) revealed that LPS also significantly increased the mRNA and protein levels of TLR4, IDO1, and KMO (Figures 6C–E,G–J). There was no difference in KAT2 among those groups (Figures 6F,G,K). Furthermore, ELISA demonstrated a concomitant increase in the levels of QUIN, a downstream KP metabolite (Figure 6B). Critically, TAK242 administration attenuated these LPS-induced effects (Figures 6B–K), mimicking the protective outcomes observed in our immunofluorescence studies (Figure 5). Collectively, these results indicate that pharmacological inhibition of TLR4 can mitigate microglial activation and the associated induction of the KP, providing insight for future therapeutic development.

## 4 Discussion

**FIGURE 7:**
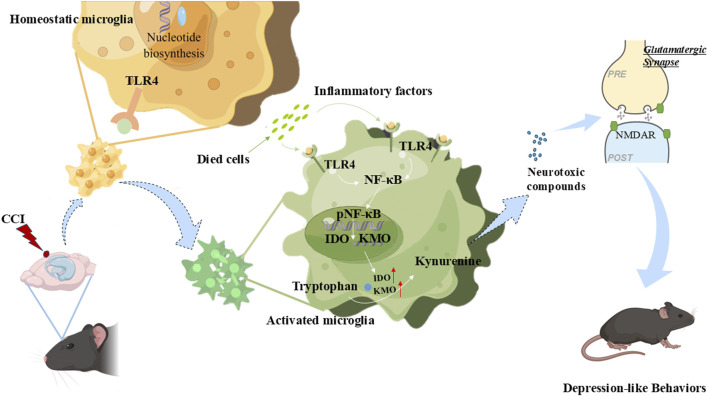
The scheme of TLR4 medicates post-TBI depression. By elucidating that chronic TBI-induced depressive behavior is driven by a TLR4–KP–QUIN axis—where TBI activates TLR4 signaling, upregulates kynurenine pathway enzymes IDO and KMO, and promotes quinolinic acid production—we integrated TLR4 signaling and KP dysregulation into a unified mechanism, thereby providing a foundation for novel immune-metabolic therapies. PRE and POST: pre- and post-synaptic.

Building on prior research (Wang B. et al., 2019), this study elucidates the role of TLR4 in chronic TBI-induced depressive behavior and its mechanism of action via kynurenine pathway activation (Figure 7). Specifically, TBI triggers TLR4 signaling, upregulating the KP enzymes IDO and KMO, which drive the production of the neurotoxic metabolite quinolinic acid (Figure 7). This TLR4–KP–QUIN axis advances our etiological understanding of post-TBI depression and integrates two major research domains—TLR4 signaling and KP dysregulation—providing a mechanistic foundation for novel immune-metabolic therapies.

Our study demonstrated that mice developed depression-like behaviors—characterized by prolonged FST immobility and reduced SPT preference—specifically at 28 days post-TBI. Although there are many methods to detect depression, only the two most commonly used methods used in this study are also applied in many studies (Chen Y. et al., 2023; Zhang B. et al., 2025). This indicates that MDD manifests as a late sequela of TBI, aligning with findings by Laaksonen et al. (Laaksonen et al., 2025), who reported stabilization of TBI-induced behavioral deficits (e.g., increased tail suspension immobility, decreased sucrose preference reflecting anhedonia) only at 4 weeks post-injury (Miao et al., 2025; Zhou et al., 2024). Further supporting this delayed onset, studies in female rats show increased FST immobility 6 weeks post-injury (Giacometti et al., 2022). A recent meta-analysis confirms the long-term mental health impact of TBI, reporting a pooled depression risk ratio of 2.08 in TBI patients, with elevated risk persisting for years or decades. Compared to non-TBI populations, TBI patients exhibit a continuously increasing depression risk within the first decade post-injury (Dehbozorgi et al., 2024; Delmonico et al., 2022; Passler et al., 2022). Using combined behavioral assessments (FST, SPT, OFT) and control reference values, we determined a post-TBI MDD incidence of ∼28% in our model. This aligns with the 37% incidence reported by Zhou et al. (2024) in a comparable model, supporting our approach. Divergent rates (e.g., ∼25% reported by Stein et al. (2019)) likely reflect differences in injury severity, animal strain, or assessment timing. Clinical meta-analyses indicate that ∼27% of TBI patients are diagnosed with MDD/dysthymia, while 38% report clinically significant depressive symptoms. Within 1-year post-TBI, 25%–50% of patients experience depression (Stein et al., 2019; Uiterwijk et al., 2022). Collectively, preclinical and clinical evidence establishes a post-TBI depression incidence range of 25%–50%.

We demonstrated that TLR4 activation contributes to post-TBI depression, and its pharmacological inhibition alleviates depressive-like behaviors. Specifically, we observed increased hippocampal TLR4 protein/mRNA levels and proteomic evidence of TLR4 pathway activation post-TBI. Critically, administration of the specific TLR4 inhibitor TAK-242 significantly ameliorated depression-like behaviors, confirming TLR4’s pathogenic role and therapeutic potential (Shirayama et al., 2022). While direct evidence linking TLR4 to depression in this TBI model was previously lacking, studies in other depression models—including chronic social defeat stress (CSDS), LPS-induced inflammatory depression, and corticosterone-induced depression—consistently report hippocampal TLR4 upregulation and its contribution to depressive behaviors (Wang Y. X. et al., 2019; Zhao et al., 2019). Notably, genetic ablation of TLR4 confers resistance to depression-like behaviors in chronic stress models (Wang et al., 2025; Wu et al., 2023). Reinforcing our findings, specific pharmacological inhibition of TLR4 also ameliorates depressive symptoms in post-stroke depression models (Abdul et al., 2019; Jiang et al., 2025). These results highlight TLR4’s broad therapeutic relevance. Enhanced expression of TLR4 pathway genes in peripheral blood monocytes from MDD patients further supports TLR4 inhibition as a promising broad-spectrum strategy for both post-TBI and idiopathic depression (Hung et al., 2016).

Regarding the mechanism, we propose that TLR4 activation modulates the KP, increasing production of the neurotoxic metabolite QUIN, thereby influencing neuronal excitability and survival. QUIN’s neurotoxicity is well-established in neurodegenerative models (e.g., Huntington’s, Alzheimer’s disease), where it damages neurons and oligodendrocytes (Song et al., 2011). This supports a conserved TLR4–KP–QUIN axis across neurological disorders. QUIN is also implicated in depression pathogenesis: elevated levels enhance glutamatergic neurotransmission, correlating with depressive behaviors (Autry et al., 2011; O’Connor et al., 2009). Clinically, QUIN is significantly increased in the CSF of MDD patients and correlates with symptom severity. Arnone et al. (2018) further reported markedly increased hippocampal QUIN post-TBI in mice (Arnone et al., 2018; Slavich and Irwin, 2014). Collectively, this evidence suggests TLR4 promotes post-TBI depression by driving QUIN production via the KP, though causal validation is needed.

While TLR4 is expressed on microglia and astrocytes (Lee et al., 2019), our study specifically observed its significant upregulation in microglia post-TBI, implicating microglial activation in post-TBI depression. Crucially, IDO1 and KMO upregulation are primarily attributed to activated microglia, while KAT2 activity (involved in glutamate homeostasis) is largely astrocyte-regulated. Substantial evidence confirms IDO1 enrichment in microglia/infiltrating myeloid cells, predominant KMO expression in microglia, and primary KAT2 localization to astrocytes. This cellular specificity explains the predominant roles of IDO1/KMO in microglia and KAT2 in astrocytes (Huang et al., 2020; Kennedy et al., 2017). Consequently, TLR4 inhibitors likely act primarily on microglia, suppressing IDO1 and KMO expression within these cells, with limited effects on astrocyte-dominated KAT2. Therefore, we put forward a consistent hypothesis: after TBI, hippocampal TLR4 is activated by DAMPs, and IDO1 and KMO are upregulated. This drives the accumulation of neurotoxic metabolites (such as QUIN), potentially impairing the function of nearby neurons and astrocytes, and eventually leading to depression.

While this study elucidated the TLR4–KP pathway in post-TBI depression, limitations remain: 1) We cannot definitively exclude TLR4 involvement in other TBI complications (e.g., AD-related cognitive impairment, PTSD fear memory); 2) TLR4’s broad cellular expression hinders full dissection of cell-specific functions and intercellular crosstalk—a persistent methodological challenge, despite existing microglia-specific conditional knockout models; 3) KP pathway contribution was not validated independently of TLR4’s inflammatory role; 4) Under certain conditions, KP activation/IDO1 expression can be driven by alternative pathways (e.g., TLR2, NLRP3 inflammasomes, IRF3 signaling); 5) Other key brain regions, including the prefrontal cortex and amygdala, were not included in the present study; 6) The role of gender-related research in KP difference between neuroinflammation and other diseases/disorders after TBI. Further investigation is needed to define the therapeutic efficacy and optimal treatment window for TLR4-targeted interventions.

## 5 Conclusion

This study established the temporal pattern and incidence of depression following TBI in mice and demonstrated that TLR4 mediates post-TBI depression, highlighting its therapeutic potential. We further elucidated that TLR4 drives depressive phenotypes by activating the KP, thereby increasing production of the neurotoxic metabolite QUIN (Figure 7). These findings identify novel therapeutic avenues for modulating TLR4 via metabolic pathways and provide a mechanistic framework for understanding post-TBI depression.

## Data Availability

The raw data supporting the conclusions of this article will be made available by the authors, without undue reservation.

## Supplementary material

SUPPLEMENTARY FIGURE 1 modified Neurological Severity Score (mNSS scores) at different time post TBI. The mNSS scores of TBI mice gradually decreased after injury, with no significant difference observed between the TBI + TAK242 and TBI + Vehicle groups.

SUPPLEMENTARY FIGURE 2 Absence of depressive-like behaviors 14 days post-TBI. **(A)** Timeline of behavioral assessments: SPT, FST, OFT. **(B)** FST: No difference in immobility time (Unpaired t-test, t = 0.6085, P = 0.5465). **(C)** SPT: No reduction in sucrose preference (Unpaired t-test, t = 0.4770, P = 0.6360). **(D,E)** OFT: No differences in total distance moved (**D**: Unpaired t-test, t = 0.2680, P = 0.7900) or center duration **(E)**: Unpaired t-test, t = 0.7106, P = 0.6818). (ns: no significance; n = 20 per group).

SUPPLEMENTARY FIGURE 3 Microglial activation (IBA1 IF) in hippocampus 28 days post-TBI and TAK242 effects. (The yellow arrows indicated microglia; n = 3 per group; scale bar = 20 µm).

SUPPLEMENTARY FIGURE 4 IDO and KMO inhibitors attenuate TBI-induced depressive-like behaviors. **(A)** Detection methods: ELISA and Behavioral assessments: SPT, FST, OFT, and EPM. **(B)** ELISA: QUIN reduced by IDO and KMO inhibitors (Data analyzed by one-way ANOVA with Tukey’s test, F_(2, 15)_ = 6.639 P = 0.0086, n = 6, 6, 6, **P < 0.01). **(C)** SPT: Increased sucrose preference (F_(2, 35)_ = 2.673, P = 0.0831. **(D)** FST: Decreased immobility time in IDO inhibitors mice (F_(2, 35)_ = 5.258, P = 0.0101). **(E,F)** The prevalence of depression-like behavior in TBI mice treated with IDO1/TDO-in-4 and GSK180 was 14.29% and 7.14%, respectively. **(G)** ELISA: Administration of IDO and KMO inhibitors modulated QUIN levels in TBI mice (Data analyzed by one-way ANOVA with Tukey’s test, F_(2,12)_ = 13.95, P = 0.0007 n = 3, 6, 6). **(H,I)** OFT: No differences in total distance moved **(H)**: F_(2, 35)_ = 0.1109, P = 0.8953) and center duration **(I)**: F_(2, 35)_ = 0.5047, P = 0.6080). (J-K) EPM: No changes in open arm time **(J)**: F_(2, 35)_ = 1.183, P = 0.3182) and entry in open arms **(K)**: F_(2, 35)_ = 0.1825, P = 0.8340). Data analyzed by one-way ANOVA with Tukey’s test, SPT, FST, OFT, EPM n = 13, 13, 12; **P < 0.01).
