# Supplementary figures and images for: TLR4 mediates post-traumatic depression via kynurenine pathway activation in a murine traumatic brain injury model
Source: Front Pharmacol. 2026 Apr 15;17:1744031. doi: 10.3389/fphar.2026.1744031 (PMC13125102; doi:10.3389/fphar.2026.1744031)

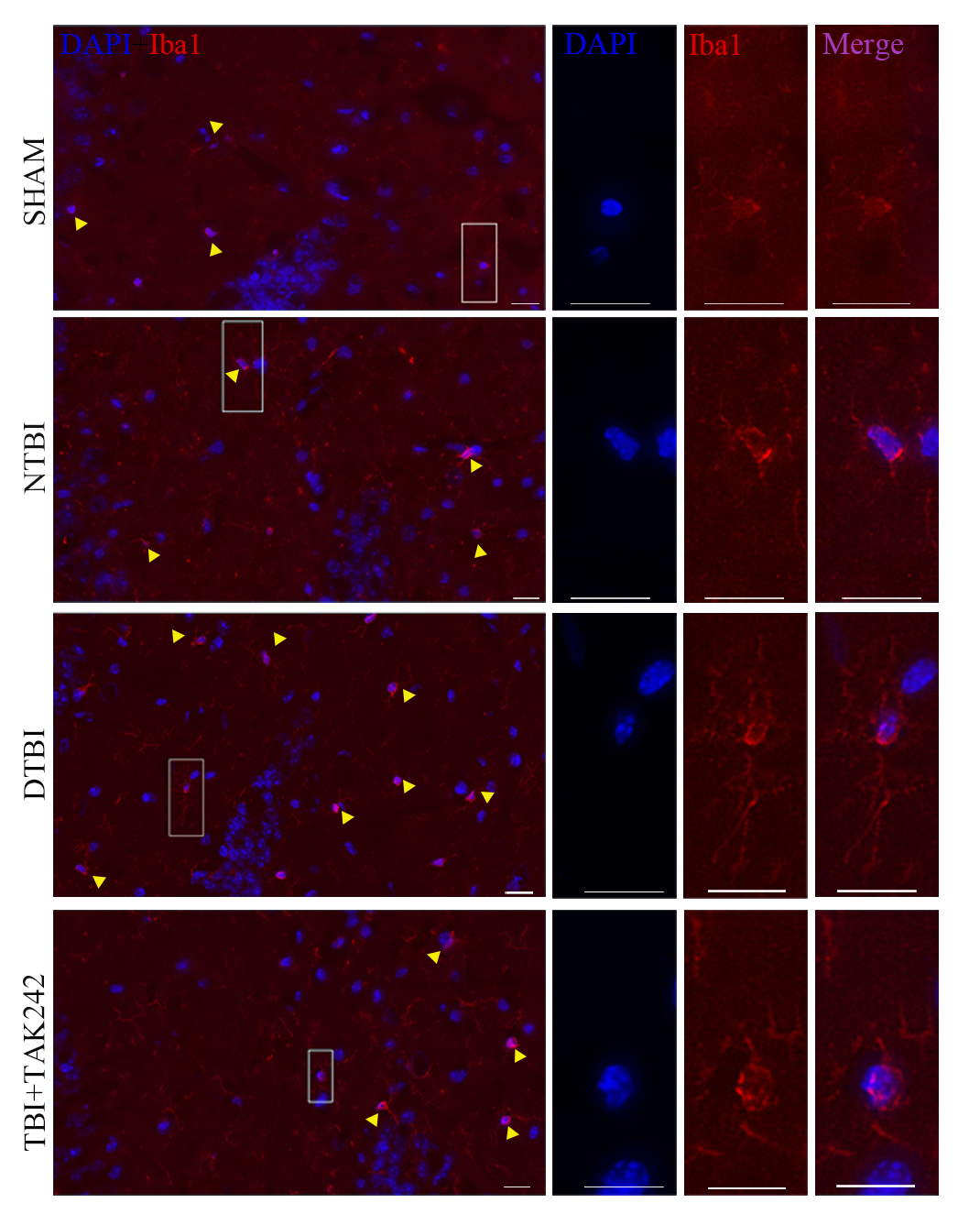

Supplement: Supplementary file 1 [file Image3.tif]

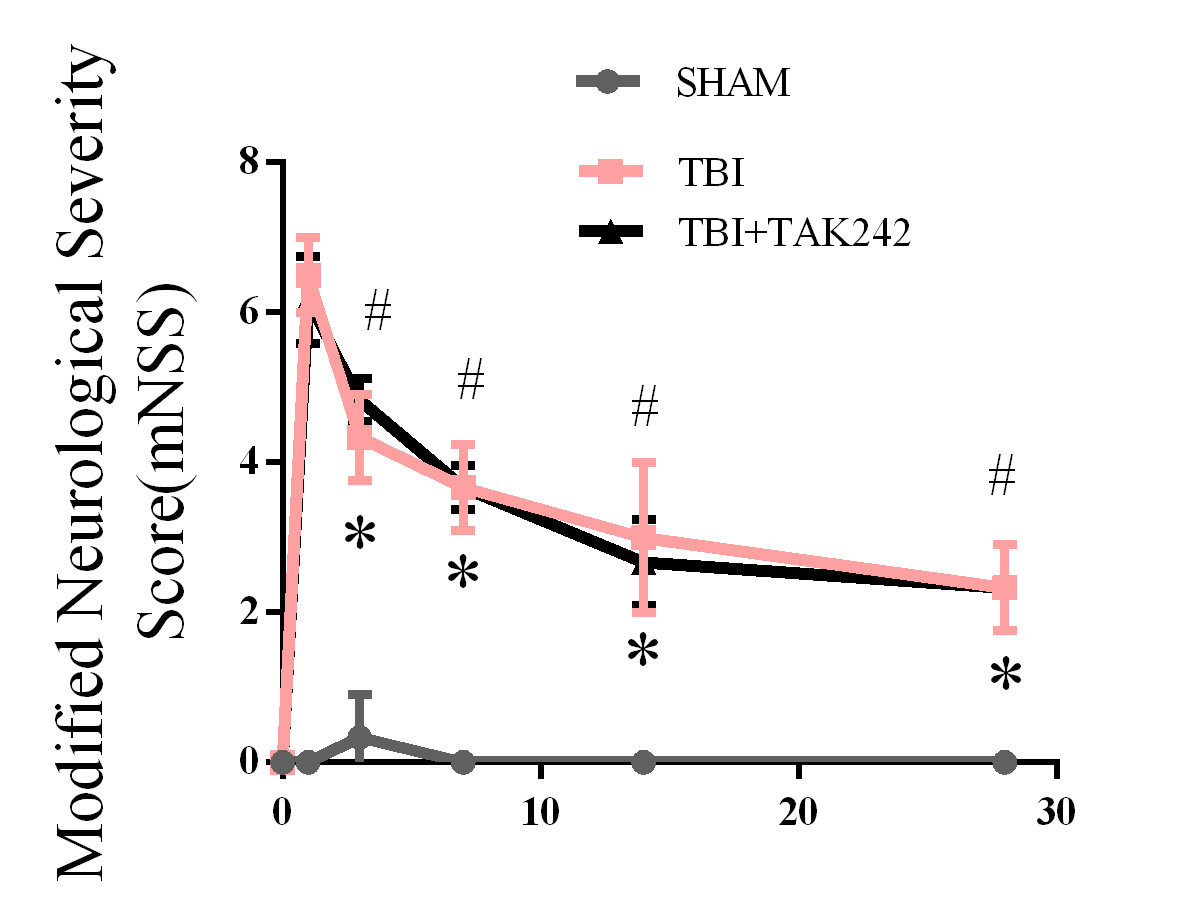

Supplement: Supplementary file 2 [file Image1.jpeg]

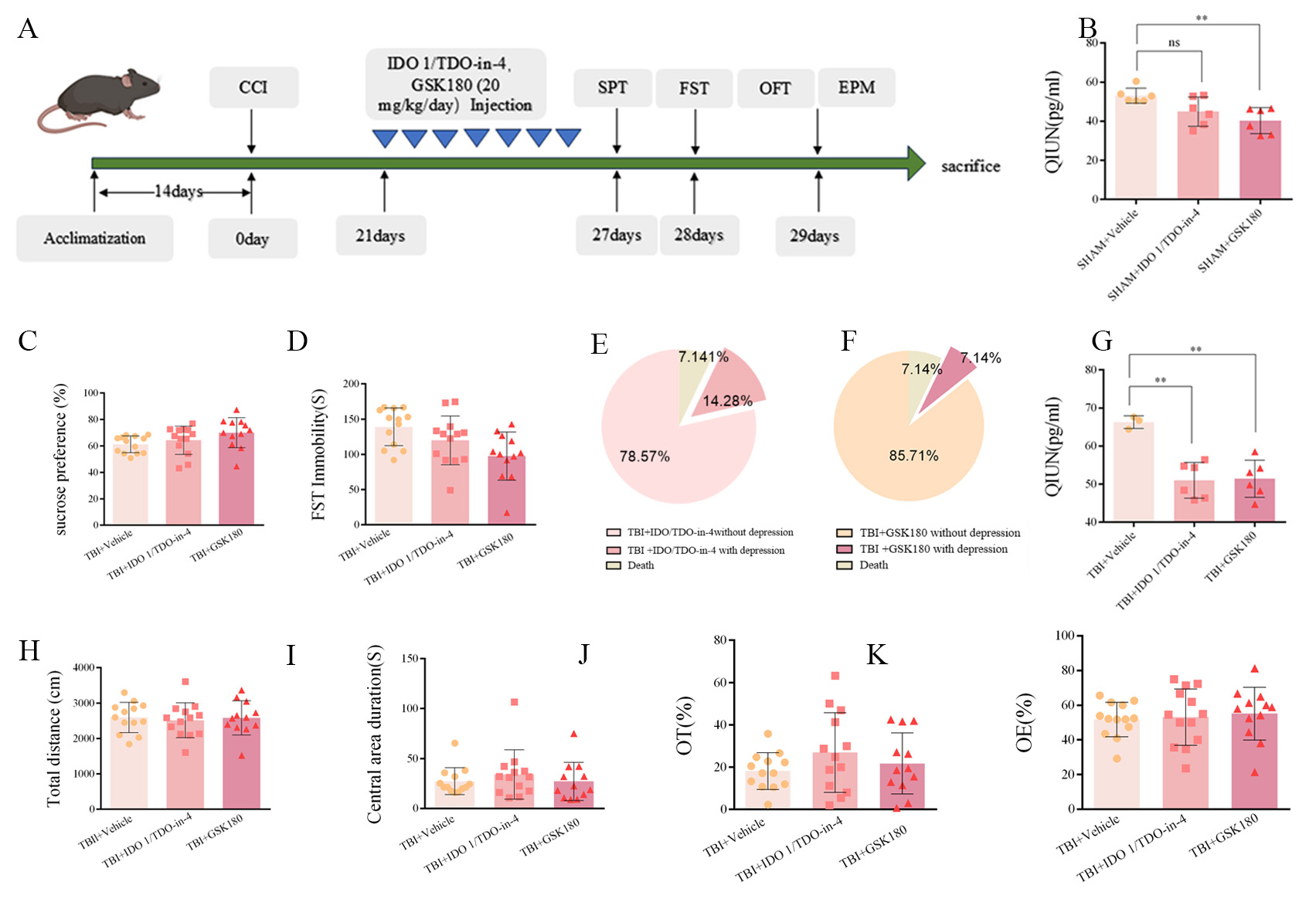

Supplement: Supplementary file 3 [file Image4.jpeg]

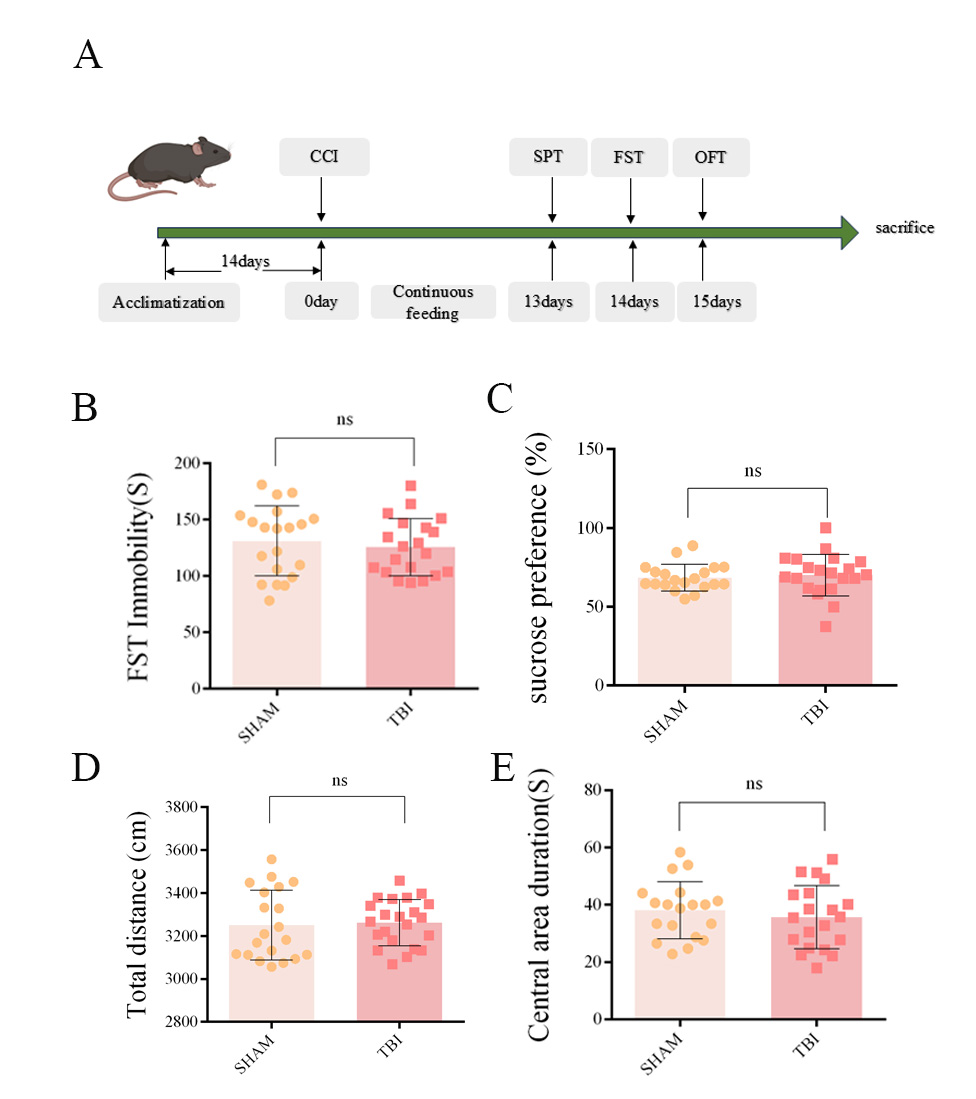

Supplement: Supplementary file 4 [file Image2.jpeg]
